# Research experiences for Canadian aspiring physicians: a descriptive analysis of medical school admission policies concerning research involvement in Canada

**DOI:** 10.1186/s12909-022-03207-y

**Published:** 2022-03-05

**Authors:** Laurie Yang, Irene Chang, Stacey Ritz, Lawrence Grierson

**Affiliations:** 1 Bachelor of Health Sciences Program, Faculty of Health Sciences, McMaster University, Hamilton, Canada; 2 Department of Pathology and Molecular Medicine, Faculty of Health Sciences, McMaster University, Hamilton, Canada; 3 Department of Family Medicine, Faculty of Health Sciences, McMaster University, Hamilton, Canada; 4 McMaster Education Research, Innovation, and Theory, Faculty of Health Sciences, McMaster University, Hamilton, Canada

**Keywords:** Aspiring physician, Medical school, Policy, Research experiences, Selection

## Abstract

**Background:**

Many aspiring physicians perceive research experience as a way to support their medical school applications; however, the importance of research experiences as articulated in medical school admission policies is unclear. This is significant since policies and other discursive signals about selection processes can influence the behaviour of aspiring physicians. The purpose of this study is to describe the ways through which Canadian medical schools articulate the importance of research experiences in publicly available policy documents.

**Methods:**

From January to June 2021, the authors reviewed publicly available selection criteria, application materials, institutional and research-related web pages associated with the 17 Canadian medical schools alongside high-level Canadian articulations of important competencies for physicians. These materials were analyzed using a qualitative descriptive approach. The authors considered concordance and/or discordance within each school’s stance on the importance of research experiences in their selection criteria and application materials.

**Results:**

Research experiences are typically not explicitly required for entry into a Canadian Doctor of Medicine (MD) program; however, there are expectations that graduating physicians should understand research. All 17 Canadian medical schools signal an appreciation for the value of research on an institutional level. Review of selection criteria and application materials show that five Canadian medical schools suggest to aspiring physicians that research experiences are important for admission and four do not suggest that research experiences are important for admission. There were both intra-institution and inter-institution discordance concerning the importance of research experiences for medical school applicant selection in one and seven medical schools respectively.

**Conclusions:**

Given the significant variance among the 17 Canadian medical schools, it is worthwhile for medical schools to evaluate their front-facing admission policies with consideration of the potential impact it might have on the behaviour of aspiring physicians, to ensure best selection of future physicians.

## Background

Entry to undergraduate medicine is highly competitive around the world. For example, the Association of Faculties of Medicine of Canada reported that only 19.60% of applicants received an offer of admission to a Canadian medical school in the 2018-2019 application cycle [1] while 42% of applicants were matriculated in American medical schools in 2020-2021 [2]. To enhance the competitiveness of their applications, aspiring physicians might seek research experiences. The perception that research experience is important for successful entry into medical school is prominent amongst aspiring physicians, as can be readily observed in online forums and websites for the aspiring physician community [3, 4]. Blogs written for aspiring physicians often reiterate this notion with statements like “*while research experience is not a requirement for admission to med school (unless you are a MD-PHD candidate), it can definitely be an advantage on your application*” [5]. As a result, a preponderance of aspiring physicians are involved in research to some degree. Past research indicates that a large number of medical students engaged in research prior to medical school [6], despite the fact that undergraduate research involvement can be time-consuming and difficult to manage alongside students’ financial and academic obligations. [7].

The degree of research involvement among aspiring physicians and perceptions of the importance of research experience for selection within the aspiring physician community may be attributed to medical schools themselves. Generally speaking, the Theory of Policy suggests that policies are an important tool through which institutions can influence the behaviour of a target population [8]. This can be exemplified in a public health context, where modest changes in public policies result in significant changes in health behaviours [9]. This is also true in medical school admissions. Past studies confirm the impact of admission policies on student behaviour. Lin and colleagues [10] determined that the aspiring physician’s narrative is shaped by their understanding of the expectations of the medical school admissions process. That is, there may be a hidden curriculum in admissions processes [11, 12], which leads applicants to model their behaviours based on their perceptions of what is valued in the selection process [13]. Although some describe this pejoratively as applicants attempting to ‘game the system,’ this is in fact very rational behaviour for aspiring physicians given the limited number of spots available, and the lack of control they have over the parameters by which they will be judged.

To understand the relevance of the theory of policy to the experiences of medical school applicants with respect to research engagement, we need to determine whether there is a consistent policy message that is being conveyed to candidates. Although research is important for healthcare delivery [14], it is unclear the degree to which applicant research experiences serve as a uniquely valuable demonstrator of commendable qualities, whether at all or relative to other experiences. At present, it is also unclear the degree to which formal selection criteria and admissions policies articulate the value of research experience and how this might be influencing aspiring physician behaviour as they prepare their applications. Accordingly, we present here an analysis of publicly available Canadian medical school admission policies pertaining to research experiences and reflect our findings against the formal competencies that medical students are expected to develop prior to graduation and statements concerning the value of research activity within training institutions. In doing so, our goal is to generate a better understanding of how research experiences are emphasized within publicly available medical school policy documents; directly or indirectly related to admissions.

## Methods

### Purpose

This study was a qualitative descriptive analysis that used descriptive document coding to examine how the importance of research experiences is articulated in publicly available policy documents from the 17 Canadian medical schools.

### Research team

As a part of our commitment to reflexive research, our team acknowledges our positions and perspectives. Our team consists of four researchers (IC, LY, SR, LG). IC and LY are undergraduate students who identify as women and who are aspiring physicians. The research began as an undergraduate course project. Upon completion of the course, IC and LY were hired and paid for their work as research assistants. SR and LG are education scientists with faculty positions at McMaster University with experience and expertise in the methodology used.

### Data collection

We reviewed four types of publicly available documentation with the potential to elucidate the relevance of applicant research experiences to medical school selection practices in Canada. Our research team extracted documents from web pages in either English or French between January 2021 and June 2021 for analysis.

We first reviewed a set of documents consisting of pan-Canadian articulations of the competencies required for admission to medical school, expected upon graduation of medical school, and required for medical practice. This included a collection of documents describing the technical standards for students of MD programs [15–26] which, for the purposes of this paper, are referred to collectively as “Essential Skills”. We also included the Royal College of Physician and Surgeons of Canada’s *CanMEDS 2015 Framework * [27]. Medical school committees created the Essential Skills documents as a response to legislation documenting requirements of accessibility for people with disabilities. As such, the Essential Skills documents are not meant to describe idealistic statements about the capacities of the physician but rather to firmly state what can and cannot be accommodated within the role of a physician. On the other hand, the Royal College of Physician and Surgeons of Canada created the CanMEDS framework to inspire curriculum and training for physicians, with the aspiration that physicians should be more than simply individuals who can treat and diagnose illness [28]. With this context in mind, we anticipate that research experience will be conceptualized differently in these documents.

To elucidate latent messages concerning research experiences (i.e., pertaining to the “hidden curriculum” of admissions), we also sampled institutional values concerning research that may not specifically pertain to medical school admissions [12]. We determined institutional values concerning research via: review of program values, missions, and vision statements; program information (or “About”) statements; and explicit statements about program stances on research on the medical school research landing page. All of the aforementioned were found on the public websites of the 17 Canadian medical schools [29–45]. In the case that undergraduate medical education-specific pages were absent, our team reviewed the relevant School of Medicine page instead. If the School of Medicine page was also absent, the Faculty of Medicine page was reviewed.

The final set of documents consisted of formal statements of medical school selection criteria and materials that support aspiring physicians in the development of their applications to medical school. We reviewed medical school selection criteria for the 17 Canadian medical schools from program websites [29–45]. This included review of the criteria, requirements, expectations, and/or traits that Canadian medical schools state as explicitly relevant for their selection decisions. We also reviewed application materials, which consist of (1) descriptions of the application (2) forms that applicants must submit to apply to the medical school. These were extracted from each of the Canadian medical school websites as well as the Ontario Medical Schools Application Service (OMSAS) website, a portal used by aspiring physicians for application to Ontario medical schools. With respect to selection criteria and application materials, we only considered documents pertaining to admissions for Canadian undergraduate or Collège d’enseignement général et professionnel (CEGEP) applicants; however, our analysis was mindful of any credit or advantage conferred to applicants with graduate degrees. We excluded any admission policies relating to non-Canadian applicants.

Throughout this paper, we refer to medical schools by the name of the university. For instance, we will use the “University of Manitoba” when referring to the “University of Manitoba Max Rady College of Medicine’’.

### Data analysis

We constructed this research as a document analysis, drawing upon procedures described by O’Leary [46]. Four reviewers (IC, LY, SR, LG) participated in the coding process. All four reviewers met initially to review the methodology of qualitative description. Our analysis of sampled documents involved a qualitative descriptive approach [47, 48]. First, to establish data immersion, members of our team (IC and LY) read and re-read the compiled data individually to gain familiarity with the information. During this process, analytic memos concerning coder insights and ideas were generated [49]. We then used open coding, which was conducted with specific consideration for statements relevant to the research question. This was done in order to describe the emphasis, contemplation, and inclusion of research experience within the four types of reviewed documents. During the coding process, textual components of selected documents were coded. Images and graphics in the documents were excluded.

Once the initial round of open coding was completed, IC, LY, SR, and LG convened over several meetings to review the codes and to develop a codebook. The initial version of the developed codebook was piloted by IC and LY on document excerpts. All reviewers re-convened for revisions of the codebook.

After codebook development, IC and LY applied codes to all data in an iterative process, meeting regularly throughout. Upon completion, SR and LG reviewed all codes. After, we engaged in a process of analysis which determined within each medical school whether selection criteria and application materials were concordant or discordant with respect to their intimation of the importance of research for candidate selection. They were considered concordant if they expressed similar views regarding the importance of research and research experience and considered discordant if they express differing views regarding the importance of research and research experiences. Cases where there was no mention of research experiences were treated as suggestive that research experiences were *not* important for selection. If there was discordance on the importance of research and research experiences within the selection criteria or application material of one school, they were deemed discordant. Our team (IC, LY, LG, and SR) met periodically following each cycle of coding to compare and discuss results and resolve any inconsistencies or discrepancies.

## Results

### Canadian articulations of the importance of research experiences for aspiring physicians and physicians

This portion of the analysis provided insight into the ways in which research, research skills, or research experiences are described in policy statements that define the essential skills for aspiring physicians and competencies for practicing physicians. Our analysis revealed that the Essential Skills document makes no mention of research competence when describing the competencies required for entry and success in medical school [15–26]. Mentions of research skills are stated more clearly within the 2015 CanMEDS framework [27], which emphasizes that “*physicians should not only participate in research but also are involved in the dissemination of research findings*” (p. 11). Key competencies in the framework uphold that statement. For instance, one key competency is to “*contribute to the creation and dissemination of knowledge and practices applicable to health*” (p. 25) and to “*integrate best available evidence into practice*” (p. 25) [27]. Overall, these policy documents suggest that although research experiences are not *essential* for aspiring physicians, physicians should eventually have the skills to engage in research and to understand research evidence.

The importance and mentions of research or research experiences within the 17 Canadian medical schools across statements of institutional values and selection criteria, and application materials are summarized in Table 1.

**Table 1:** Research / research experiences within Canadian medical schools’ institutional values, selection criteria, and application materials

	Institutional values			Selection criteria		Application		
						Suggests research experiences *are* important for selection to medical school		Suggests research experiences are *not* important for selection to medical school
	Research as a value to the physician/learner	Research as a value to society	Research as an indicator of prestige	Suggests research experiences *are* important for selection to medical school	Suggests research experiences *are not* important for selection to medical school	Exclusive section(s) to describe research experiences	Section(s) prompt description of research experiences via examples	No section to describe research experiences
Dalhousie University	✓	✓	✓		✓	✓		
McGill University	✓	✓	✓		✓	✓		
McMaster University	✓				✓			✓
Memorial University of Newfoundland		✓			✓		✓	
Northern Ontario School of Medicine	✓	✓	✓	✓		✓		
Queen’s University	✓	✓	✓	✓		✓		
University of Alberta	✓	✓	✓		✓		✓	
University of British Columbia	✓	✓	✓	✓		✓	✓	
University of Calgary		✓		✓		✓	✓	
University of Manitoba	✓	✓	✓	✓		✓		
University of Ottawa	✓	✓	✓		✓	✓		
University of Saskatchewan	✓	✓	✓		✓	✓		
University of Toronto	✓	✓	✓	✓	✓	✓		
Université de Montréal		✓	✓		✓			✓
Université de Sherbrooke	✓	✓			✓			✓
Université Laval	✓	✓	✓		✓			✓
Western University		✓	✓		✓		✓	
Total (/17)	13	16	13	6	12	10	5	4

### Institutional values concerning research

Review and analysis of documentation pertaining to the value that training institutions place on research highlighted that institutions hold that: research is valuable to physicians or learners; research is valuable to society; and research is an indicator of prestige. Overall, every single Canadian medical school presented statements that avowed its appreciation for research in at least one of these categories. The degree to which the importance of research to the institution was emphasized varied between medical schools. Certain medical schools invoked only one of the values of research, while other schools mentioned all three.

Most medical schools (*n* = 13) described research as a skill or practice that is of value to practicing physicians or learners (i.e., medical school students) (Table 1). Often, this was emphasised via the importance of research to medical practice. In many cases, the statement was framed in a way that encouraged medical students to engage in research. For example, the University of Toronto states that:

*As a physician with research skills, you are in the position to advance the frontiers of medical practice and improve patient care, whether discovering new treatment options, establishing the benefits of existing clinical practices, or evaluating the current healthcare system for potential policy changes. That is why research experience is favoured by hospital or residency admission committees and can help open a broader spectrum of career avenues*. (University of Toronto, “Research Opportunities”) [37].

Nearly all medical schools (*n* = 16) referenced research as something of value to society (Table 1). Research is largely considered a tool that can be used to improve healthcare and well-being on both an individual and community level. For example, University of Calgary states: “*A research intensive medical school translates to better health care for our community*” (University of Calgary, “Research”) [43].

Over half of the medical schools (*n* = 13) referenced research as an indicator of the institution’s prestige (Table 1). Some schools emphasized their international or national research contributions. For example, McGill University claims to be “*the leading medical research university nationally, and … renowned for its excellence in the health sciences worldwide*” (McGill University, “About the Faculty of Medicine and Health Science”) [31]. The University of Ottawa states that its “*Faculty of Medicine has a long history of conducting both basic and clinical research of the highest quality*“(University of Ottawa, “About Us”) [44]. These kinds of statements equating research prowess with excellence in health sciences were extremely common among Canadian medical schools.

### The value placed on research experiences in selection criteria and application materials

We present our analysis of selection criteria and application materials together. This allows us to describe the way that research experiences are valued within these articulations of admission policy, while also permitting us the opportunity to highlight the degree of concordance or discordance that may exist between them at one school.

Our analysis revealed that only five Canadian medical schools have selection criteria and application materials that both suggest that research experiences are important for admissions (Table 1). Within the selection criteria, these schools explicitly describe the ways in which research experiences are part of their evaluation and file review process. For example, University of Calgary indicates that it “*will review the entire [application] file and will assign scores for: […] Intellectual curiosity, scholarly activity, and research (10%).*” (University of Calgary, “2020-2021 Applicant Manual”) [43]. The application materials for these five schools includes sections where applicants must describe their research experiences and/or list their research publications and presentations. In addition, two of these schools also prompted research experiences as an example of an activity that can be described in a certain section. For instance, the University of Calgary stated:

*Top 10 Experiences*

*Applicants are given the opportunity to identify up to 10 activities or experiences that they feel are sufficiently important as to define them as individuals. These may be employment or volunteer experiences, life experiences, awards, educational or research experiences.* (University of Calgary, “2020-2021 Applicant Manual”) [43].

On the contrary, four Canadian medical schools suggest that research experiences are not important for selection, and this is represented in both their selection criteria and application (Table 1). Out of these four, three are located in Quebec. While each of these schools iterate the importance of research at an institutional level, comments regarding the importance of research experiences in the medical school’s selection criteria and supplementary application are absent. It is noteworthy that the three Quebec schools belonging to this category have a separate evaluation and application process for streams of candidates who have completed graduate degrees in addition to undergraduate or CEGEP training, in which case research experiences are emphasized as important.

A moderate number (*n* = 7) of Canadian medical schools fall into a third category, where their selection criteria and application materials have discordant suggestions regarding the importance of research experiences for selection (Table 1). In each case, this discordance lies within contradictions in: the selection criteria that *do no*t suggest that research experiences are required for admission; and application materials that contain sections suggesting research experience is an activity of interest to the reviewers of the application. These application sections either offer research as an example of a relevant experience applicants can include in a certain section (*n* = 3) or reflect places for the exclusive description of research experiences, either by requiring applicants to list their peer-reviewed academic publications or by requiring a declaration of a research course supervisor when available (*n* = 4). There are no schools where the reverse is true (i.e., the selection criteria suggests that research experiences are important, but the application does not). Notably, the selection criteria of 6 of the schools made no explicit statements on the importance of research experiences for selection (Table 1). The exception is Western University, which explicitly clarified that research experiences are not required for selection to medical school through the following statement: “*We are not looking for any specific extracurricular activities or experiences in our Abbreviated Autobiographical Sketch. Some of you may have done research or volunteering, some of you may not have. We also do not require any specific publications*.” (Western University, “Frequently Asked Questions”) [45]. Western University’s application materials included sections where research experiences can be listed based on the applicant’s discretion.

Lastly, one school, the University of Toronto, simultaneously suggests that research experiences are important for selection to medical school and not required for selection to medical school. This school assesses the application according to four clusters based on the CanMEDs Framework. One cluster is that of a scholar which is characterized by “*academic standing, achievements in leadership, research and social responsibility as demonstrated by (but not limited to) awards, conference presentations, publications and scholarships*.” (University of Toronto, “Non-Academic Requirements”) [37]. Despite research experiences playing a role in the school’s evaluation of applicants, another excerpt pertaining to selection claims that research experiences are not needed: “*There is nothing that you ‘need’ in the Autobiographical Sketch. You will not be penalized for interest in the clinical side of medicine, rather than research, or vice versa.”*(University of Toronto, “Frequently Asked Questions”) [37]. The application materials for the University of Toronto include an exclusive section in the Autobiographical Sketch for research.

Of particular note, although our analysis focused on policies pertaining to applicants who had completed undergraduate or CEGEP training, the admission policies associated with 9 Canadian medical schools conferred some sort of advantage to applicants with graduate degrees. Most frequently, graduate applicants would receive a bonus applied to their Grade Point Average calculation or to their overall file score. We highlight this as potentially important given that graduate degrees are often associated with a research-intensive component, such that these policies may serve to have influence on the way undergraduate and CEGEP aspiring physicians perceive the value of research experiences to the admissions process.

## Discussion

There are many instances of unclear, discordant, or absent messages from universities regarding the importance of research experiences for medical school admissions. In this regard, it is important to recognize that medical school admission policies will have intended and unintended consequences. Discordant messages may cause significant confusion for applicants, and inadvertently encourage ‘gamesmanship’ and ‘resume-padding’ activities, particularly if students perceive research experiences simply as a driver for selection outcomes rather than a means to fulfill more intrinsic motivations and interests. Such behaviour in medical students has already been documented. Among medical students, 43% indicated that their main reason for research participation was to influence entry into their residency of choice [6]. Additionally, another study found that a third of medical students who take a year off from their studies to pursue research do so primarily to increase the competitiveness of their residency applications [50].

The intended consequence of selection processes that suggest research experiences are important would ideally result in aspiring physicians who have a genuine interest and competence in research and are thus in alignment with that aspect of the CanMEDS framework. However, all aspiring physicians do not have the same access to opportunities to exhibit their merit for admission due to their different economic and social backgrounds [51]. This may be particularly important when considered alongside the way social privilege advantages those with greater access to research experiences and those able to engage in research without having to balance various responsibilities (such as employment, family support, domestic labour). As such, the markers used to evaluate aspiring physicians should closely reflect the true qualities necessary to train the physicians our societies need and encourage aspiring physicians to behave in a way that best prepares them for a future career in medicine [52].

It is noteworthy that some of the discordance may be a function of factors associated with the jurisdiction in which the medical school is located. For instance, 3 of the 4 Canadian medical schools that suggest to applicants that research experiences are not important in their selection criteria and application are located in Quebec. This might reflect Quebec’s unique CEGEP system, and consequently a distinct pool of aspiring physicians. Compared to schools outside of Quebec, these three Quebec medical schools tend to admit younger students [1] who are able to apply without an undergraduate degree. Moreover, Ontario medical schools maintain varying stances on the importance of research experiences in selection, yet collectively use OMSAS to facilitate the application process. This may be confusing to applicants, since OMSAS requests the same information from all applicants to Ontarian medical schools regardless of what materials each individual school actually considers in their selection process [53]. Some Ontario schools do not consider the OMSAS Autobiographical Sketch in their selection process at all [36, 45], while some schools use the Autobiographical Sketch as part of their evaluation [34, 37, 39, 44]. This variation may partly account for the heterogeneity between the value of research experience implied by Ontario medical schools’ selection criteria and application materials.

In completing our analysis, we are struck by the abundance of medical schools with institutional messaging that equates research prowess with excellence. We feel that aspiring physicians may pick up on that messaging and draw the conclusion that because institutions regard research prowess as an indication of excellence on an institutional level, they also regard research experiences as an indication of excellence on an individual level. That said, we recognize that research experiences are not necessarily contemplated this way when selecting aspiring physicians. Indeed, medical schools may suggest that research experiences are not important for selection because they believe that successful applicants will be taught the necessary research skills during their undergraduate and postgraduate medical training. Furthermore, the discrepancy between the institutional level descriptions of research importance and the selection and application-level descriptions of research may also reflect an attempt by medical schools to construct a more inclusive selection process that accommodates applicants with diverse experiences, backgrounds, and unequal access to research experiences. This train of thought is potentially reflected in the selection criteria of several schools, such as the University of British Columbia and Memorial University of Newfoundland, which acknowledge extraneous personal circumstances that can impact the extracurricular activities applicants are able to engage in. Whether the absence of research experiences from medical school’s selection criteria successfully ensures a more equitable selection process or rather confuses applicants is yet to be determined.

The ways in which Canadian medical schools ask applicants to describe their research experience is principally centered around research productivity metrics, described in terms of publications, presentations, awards, and grants. Given that Canadian medical schools generate hundreds of thousands in research revenue every year [1], we can speculate that a medical school’s interest in an aspiring physician’s research productivity could be partially related to the medical school’s own research goals. However, research productivity as a surrogate measurement for research experiences will have its own unintended consequences. For instance, applicants might artificially inflate their research experiences and productivity through the publication of low-impact studies [54] or engagement in predatory journals [55]. Research productivity as a surrogate for research experiences from applicants aligns with the theme of research as an indicator for prestige and excellence as constructed in this study and in previous work on the discourses found in Canadian medical school web pages [56].

Our study has a few limitations. Firstly, only the data posted publicly on the Canadian medical school websites were sampled and analyzed with respect to selection criteria and application information; resultantly, there may be more information about each medical school’s selection criteria or application that was not presented in these locations and therefore not captured in our study. Our analysis of medical school policies was limited to the most recent documents and webpages (as of the 2020-2021 application cycle, or the latest update of the webpage during time of sampling). Documents and web pages from previous years or cycles were not considered or analyzed. We recognize that medical school policies and regulations from previous cycles may have a large impact on student behaviour and understanding of medical school requirements, especially since many aspiring physicians will begin preparing for medical school admissions several years prior to applying.

### Practical implications of the study’s findings :

- Given the discordance within admission policies, medical schools should carefully appraise their admission policies for clarity and consistency.
- It is worthwhile for medical schools to consider the true importance of research experiences in the selection of aspiring physicians and the development of future health care providers, and in turn consider whether this is reflected within their admission policies.
- Discordance within medical school admission policies might not be unique to a Canadian context. Educators of other jurisdictions may wish to contemplate the implications of their admission policies concerning research experience on the behaviours of aspiring physicians in their region, asking themselves whether policies are having their intended outcomes.

## Conclusion

Within the 17 Canadian medical schools, there is a significant amount of heterogeneity in the front-facing policies regarding the importance of research experience for admission to medical school. This heterogeneity may cause confusion for aspiring physicians who are in the process of preparing for medical school applications and should be considered with respect to the intended and unintended consequences of promoting research activity amongst aspiring physicians. At this point, we can only speculate about the impact Canadian medical school admissions policies have on aspiring physician’s decisions to engage and seek research experiences; further research on the topic should seek to acquire an understanding of the importance of research experiences for selection to medical school as perceived by aspiring physicians and medical school admission committees. Data on research experience from admitted applicants could be helpful in further exploring the value of research experiences for admission to medical school.

## Data Availability

Data that support the findings of this study are publicly available on the admissions websites of all 17 medical schools in Canada. The data are cited in references: 15-26, 28-45, and 53.

## Abbreviations

MD: Doctor of Medicine
Essential Skills: Collection of documents describing the technical standards for students of MD programs
OMSAS: Ontario Medical Schools Application Service
CEGEP: Canadian undergraduate or Collège d’enseignement général et professionnel
